# Optimizing quality assurance CT schedules during adaptive proton therapy for enhanced patient care

**DOI:** 10.1002/acm2.70142

**Published:** 2025-07-13

**Authors:** Nrusingh C. Biswal, Mark J. Zakhary, Abdul K. Parchur, Ruslan Mogilnay, Matthew J. Ferris, Elizabeth M. Nichols, Matthew E. Witek, ByongYong Yi

**Affiliations:** ^1^ Department of Radiation Oncology University of Maryland School of Medicine Baltimore Maryland USA; ^2^ Maryland Proton Treatment Center Baltimore Maryland USA; ^3^ Department of Radiation Oncology Medical College of Wisconsin Milwaukee Wisconsin USA

**Keywords:** adaptive planning, brain cancer, head‐and‐neck cancer, prostate cancer, proton beam therapy, QACT

## Abstract

**Purpose:**

This study aims to optimize the frequency of routine quality assurance CT (QACT) scans in proton beam therapy (PBT) for brain, head and neck (HN), and prostate treatments. The goal is to enhance treatment efficiency without compromising treatment quality. By reducing the frequency of QACT scans, the study seeks to minimize imaging dose and better utilize patient and staff resources.

**Methods and materials:**

A retrospective, IRB‐approved review analyzed 878 QACTs from 263 patients across three anatomical sites treated with PBT at our center in 2020. The study examined QACT and replanning patterns to identify the most efficient QACT schedules. Observational and post‐implementation analysis utilized treatment data spanning from 2021 to 2022. The QACT and replanning patterns of each site were analyzed to identify the optimal frequency of QACTs.

**Results:**

Out of 205 QACTs for brain cancer patients, only five (2.5%) led to adaptive planning, indicating that a single QACT at the start of the second treatment week is optimal. In HN cases, 38 of 437 QACTs (8.7%) necessitated replanning, leading to the recommendation of an initial QACT during the first three fractions then followed by QACT for every 10 fractions. For prostate cancer, three out of 236 QACTs (1.3%) were used for adaptive planning, with the suggestion of one initial QACT during the first three fractions, and possibly an additional scan halfway through for patients with more complex treatment plans, such as involving seminal vesicles and lymph nodes and/or planned as a sequential boost.

**Conclusion:**

The retrospective analysis proposes a significant reduction in QACT frequency, which could reduce the overall QACT burden while maintaining treatment quality and accuracy. This tailored approach to QACT scheduling represents an important step in optimizing proton therapy protocols, improving patient experience, and conserving medical resources.

## 1 INTRODUCTION

Intensity‐modulated proton therapy (IMPT) with its advanced pencil‐beam scanning technology has emerged as a cornerstone in the management of various tumors, owing to its unparalleled precision in dose delivery. This precision enables the maximal concentration of radiation dose at the tumor site with a sharp distal dose fall‐off, ensuring the effective eradication of tumor cells while sparing the surrounding healthy tissues. Unlike traditional photon therapy, proton therapy's efficacy is intricately linked to patient‐specific anatomical and positioning nuances, rendering it highly sensitive to any changes in the water‐equivalent path length or alterations in the geometry and density of the tumor and adjacent organs at risk (OARs).^1^, ^2^, ^3^ These changes can significantly impact the distribution of the delivered dose, necessitating constant vigilance through quality assurance measures to adapt the treatment plan to ensure that the actual dose delivery aligns with the planned therapeutic objectives.^4^, ^5^, ^6^, ^7^, ^8^, ^9^, ^10^, ^11^, ^12^ Feasibility of online adaptation has been proposed by calculating dose on cone‐beam CT (CBCT) images and alerting clinicians when dose deviations exceed tolerances.^13^, ^14^, ^15^, ^16^, ^17^ However, it has very limited clinical use due to uncertainties in in‐room limited image quality, in‐room image‐based dose calculation, deformable image registration, contour propagation to in‐room images, etc.

The practice of employing routine quality assurance CT (QACT) scans is integral to the administration of proton beam therapy (PBT), serving as a critical checkpoint to verify the continued appropriateness of the treatment plan over the course of therapy. Through these scans, clinicians can identify and respond to significant anatomical changes that may compromise the treatment's efficacy or increase the risk to adjacent healthy tissues. The literatures point out the pivotal role of these verification scans in enabling dynamic treatment adaptation, particularly highlighting their impact in treatments involving highly variable anatomical sites such as head and neck (HN) cancers, where the propensity for significant anatomical change during treatment is higher.^18^, ^19^, ^20^, ^21^ However, the conventional approach of conducting weekly QACT scans, while comprehensive, presents considerable logistical and financial burdens to healthcare systems and can contribute to patient discomfort and increased cumulative radiation exposure. Recent studies, including those by Evans et al.,^20^ Kraan et al.,^3^ and Hedrick et al.,^12^ have brought to light the critical influence of timely QACT scans in facilitating treatment replanning, emphasizing the need for a strategic balance between rigorous quality assurance and the efficient allocation of healthcare resources.

Here, we advance the optimization of QACT scheduling by undertaking a retrospective analysis of QACT utilization and the ensuing need for adaptive replanning among patients receiving PBT for brain, HN, and prostate cancers, which are three of the five leading cases treated in our center. By dissecting the patterns of anatomical changes and their implications on treatment efficacy across these distinct anatomical sites, this research endeavors to distill a nuanced, evidence‐driven framework for determining QACT frequency. The objective is to cultivate a patient‐centered, resource‐efficient approach that harmonizes the imperative of upholding superior treatment quality with minimizing the logistical and psychological impacts of frequent QACT scans. Through a meticulous review of past interventions and outcomes, the study aims to unveil a tailored strategy for QACT frequency that not only adheres to the principles of precision oncology but also significantly enhances patient experience and optimizes the utilization of critical healthcare resources. To the best of our knowledge, no such studies address QACT patterns specific to anatomical sites to consider all patient‐related changes that might trigger replanning. We retrospectively analyzed QACT patterns and the resulting adaptive replanning of patients treated with proton beams at our institution. For brain and prostate patients, the adaptive replanning rate is quite low, while for the HN patients, the QA burden is quite significant. These anatomical sites were selected to capture a spectrum of anatomical stability and variability during treatment, facilitating the optimization of QACT schedules. Brain and prostate cancer treatments typically exhibit minimal anatomical changes, whereas HN treatments often demand frequent adjustments due to significant anatomical variability. Here, we included all patients treated with PBT for brain, HN, and prostate cancers at our institution in 2020—three of the five most commonly treated anatomical sites—without applying additional exclusion criteria. This inclusive approach enabled a comprehensive assessment of clinical QACT utilization and adaptive replanning patterns across these distinct clinical scenarios. Hence, we have chosen to investigate these three sites in our initial phase.

## 2 MATERIALS AND METHODS

### 2.1 Patients

In this study, approved by the Institutional Review Board (IRB), we conducted a comprehensive review of patients who underwent PBT across three major anatomical sites at our facility in 2020. During this timeframe, 263 patients received treatments, with 40 patients (representing 5.2% of the total) undergoing replanning based on their QACT scans. In total, 878 QACT scans were carried out, with 46 of these scans leading to adaptive planning modifications. Detailed patient information and the distribution of QACTs across the various anatomical sites are presented in Table 1. Furthermore, treatment data spanning from 2021 to 2022 were analyzed for observational and post‐implementation assessment purposes.

**TABLE 1:** Patient demographics, cumulative QACT scan counts, and adaptive replanning summary for the three anatomical sites.

Anatomic sites	No. of patients	Median age, y (range)	No. of QACTs (avg./patient)	No. of patients replanned (%)	No. of replans (% QACTs used for replanning)
Brain	82	52 (5–81)	205 (2.5)	4 (4.8%)	5 (2.4%)
HN	97	62 (21–90)	437 (4.5)	33 (34.0%)	38 (8.7%)
Prostate	84	69 (50–82)	236 (2.8)	3 (3.6%)	3 (1.3%)

Average QACTs per patient reflect site‐specific variability in scan frequency.

### 2.2 Treatment planning

Simulation scans were performed using a CT scanner (Siemens Somatom Definition Edge, Germany) with patients in the head‐first supine position. Brain and HN patients were immobilized with a BoS Headframe (CQ Medical, Pennsylvania, USA) and 3‐point mask, and 5‐point mask, respectively. Prostate cancer patients were scanned on a vacuum cushion (VacQfix, CQ Medical, Pennsylvania, USA) with a full bladder protocol. Axial images were acquired every 1.5 mm from the top of the skull superiorly to shoulder level for brain patients, from the top of the skull to one vertebral body below the carina inferiorly for HN patients, and every 3.0 mm from L3 inferiorly to the mid‐thigh for prostate cancer patients. Tentative isocenter positions were marked with radiopaque markers. Structure delineation and treatment planning were performed using the RayStation 8A (RaySearch Laboratories, Stockholm, Sweden) treatment planning system (TPS). The RayStation TPS's Monte Carlo–based dose optimization and dose calculation engine were utilized to generate the treatment plans.

#### 2.2.1 Brain

The beam direction strategy typically included wide hinge angles to minimize overlap with vital OAR. Beam angles were optimized to reduce the dose to critical OARs (optical structures and brainstem). The optimization of the beams was executed through a single‐filed optimization (SFO) technique, aiming for the coverage objectives of encompassing 100% of the gross tumor volume (GTV) and 95% of the clinical target volume (CTV) with at least 100% of the intended dose, simultaneously keeping the dose to OARs below the threshold set by the guidelines of our institution. Spot weights ranged up to 30 monitor units (MU) per spot, with higher weights avoided in areas near critical OARs. Range shifters of 2–5 cm were employed for superficial brain targets and no shifter was for deeply located tumors. The spot spacing and energy spacing of the beams were adjusted within 0.7–1.2 σ, ensuring the exclusion of 110% hotspots specific to each beam. Optimization and planning for both targets and OARs were meticulously carried out accounting for a 3 mm setup margin and a 3.5% range uncertainty. Robust evaluation was performed using 3mm setup uncertainty and 3.5% range uncertainty.

#### 2.2.2 HN

The HN subtypes treated at our institution were the oropharynx, nasopharynx, paranasal sinus, and unilateral targets (e.g., parotid gland, skin, tonsil, etc.). To enhance the robustness of dose distribution, our institute mandates that each segment of the target must receive exposure from at least two beams, with no single beam delivering more than 70% of the intended dose. These plans were fine‐tuned using the Multi‐Field Optimization (MFO) strategy to meet our coverage objectives: ensuring 100% of the GTV and 95% of the CTV received at least the full prescribed dose, while keeping the dose to OARs within safe limits as per our institution's guidelines. The coplanar beams were set up at gantry angles with one beam directly at 0° and the others ranging from 150° to 210°, to include posterior‐anterior (PA) and posterior–oblique orientations.^22^

These plans underwent robust optimization, focusing on the CTVs as the primary target volumes, and accounted for uncertainties of 3‐mm in lateral, longitudinal, and vertical dimensions, as well as a 3.5% uncertainty in range. This process involved the consideration of 12 worst‐case scenarios, insisting that the minimum dose to 95% of the CTV (D95%) exceed 95% of the prescribed dose under the most adverse setup (involving Cartesian shifts but excluding rotation) and range conditions. Independent optimization of each beam was not applied. The spot and energy layer spacings varied based on the usage and type of range shifter in the plan. When a 3‐cm range shifter was employed, adjustments were made so that both spot and layer spacing remained within a 0.5–0.9 σ. Critical serial OARs such as the spinal cord, brainstem, and optic pathways were specifically targeted for protection during robust optimization. The selection of beam angles was carefully made to disperse the end‐of‐range effects across OARs, minimizing the impact of distal relative biological effectiveness.

#### 2.2.3 Prostate

Treatment typically targeted the prostate, seminal vesicles (SVs), and lymph nodes. Depending on individual patient needs, treatment plans varied between sequential and simultaneous integrated boost (SIB) techniques. In cases where patients had hip implants, these implants were digitally adjusted to reflect their actual material composition in the planning software. Any imaging distortions caused by these implants were corrected to represent the surrounding tissue accurately, usually muscle or fat. For targeting the prostate and SVs directly, two parallel opposing bilateral beams were generally employed. When treatment plans included lymph nodes, an additional beam from the back (posterior) was added to the two lateral beams. Patients who had received prostate seed implants previously were typically excluded from this treatment protocol. The standard setting for energy layer and spot spacings was set to 1 σ. Treatment plans were precisely tailored using an MFO method for cases involving the prostate and lymph nodes, and an SFO strategy for solely the prostate or the prostate combined with SVs, factoring in a 5 mm setup variability and 3.5% uncertainty in radiation range. To assess the strength and reliability of each plan, 21 different scenarios were tested, incorporating variations of up to ± 5 mm in setup positioning and ± 3.5% in radiation range, ensuring that in the least favorable scenario, at least 95% of the clinical target volume (D95%) received no less than 95% of the intended dose.

### 2.3 Treatment delivery

#### 2.3.1 Brain treatment delivery

The treatments were administered using a ProBeam system (by Varian Medical Systems, Inc., Palo Alto, California, USA) equipped with a 6‐degree‐of‐freedom (DOF) robotic treatment couch. Adhering to our institution's protocol, the patient alignment process begins with a kilovoltage (kV) to kV imaging for skeletal structure alignment. This is followed by a CBCT for both skeletal and soft tissue verification to ensure precise alignment with the treatment plan as depicted in the planning CT images. The alignment process concludes with another set of kV to kV images to confirm the accuracy of the treatment setup.

#### 2.3.2 HN treatment delivery

For HN patients, the setup procedure starts with kV to kV imaging to align the bony structures. This is followed by a CBCT scan to confirm the alignment of both the bone and soft tissues with the treatment plan outlined in the planning CT. Treatment verification is then conducted with an additional kV to kV imaging at the beam's eye view. The necessity of CBCT for each treatment fraction is evaluated on a case‐by‐case basis. If the treatment field's couch angle is altered during therapy, a kV image is acquired to facilitate couch adjustment for accurate treatment delivery.

#### 2.3.3 Prostate treatment delivery

Prostate treatments commence with initial patient setup through kV to kV imaging for alignment based on the pelvic bones. This is followed by a CBCT to verify the alignment of the bony structures and to assess the prostate and rectum interface, as well as the filling states of the bladder and rectum.

### 2.4 Quality assurance CT (QACT) scans

The assessment of QACT scans for dosimetric variations is integral to our treatment process. The frequency and necessity for QACTs are dictated by departmental guidelines, which consider clinical aspects such as histological type, stage of cancer, and any concurrent systemic treatments. For each anatomical site, we meticulously reviewed QACT and replanning data for each patient to deduce an optimal QACT schedule that aligns with the actual replanning events observed. We also evaluated potential delays and the risk of missing a necessary replan associated with both the current and proposed reduced QACT schedules.

## 3 RESULTS

### 3.1 Brain cancer treatment

**FIGURE 1:**
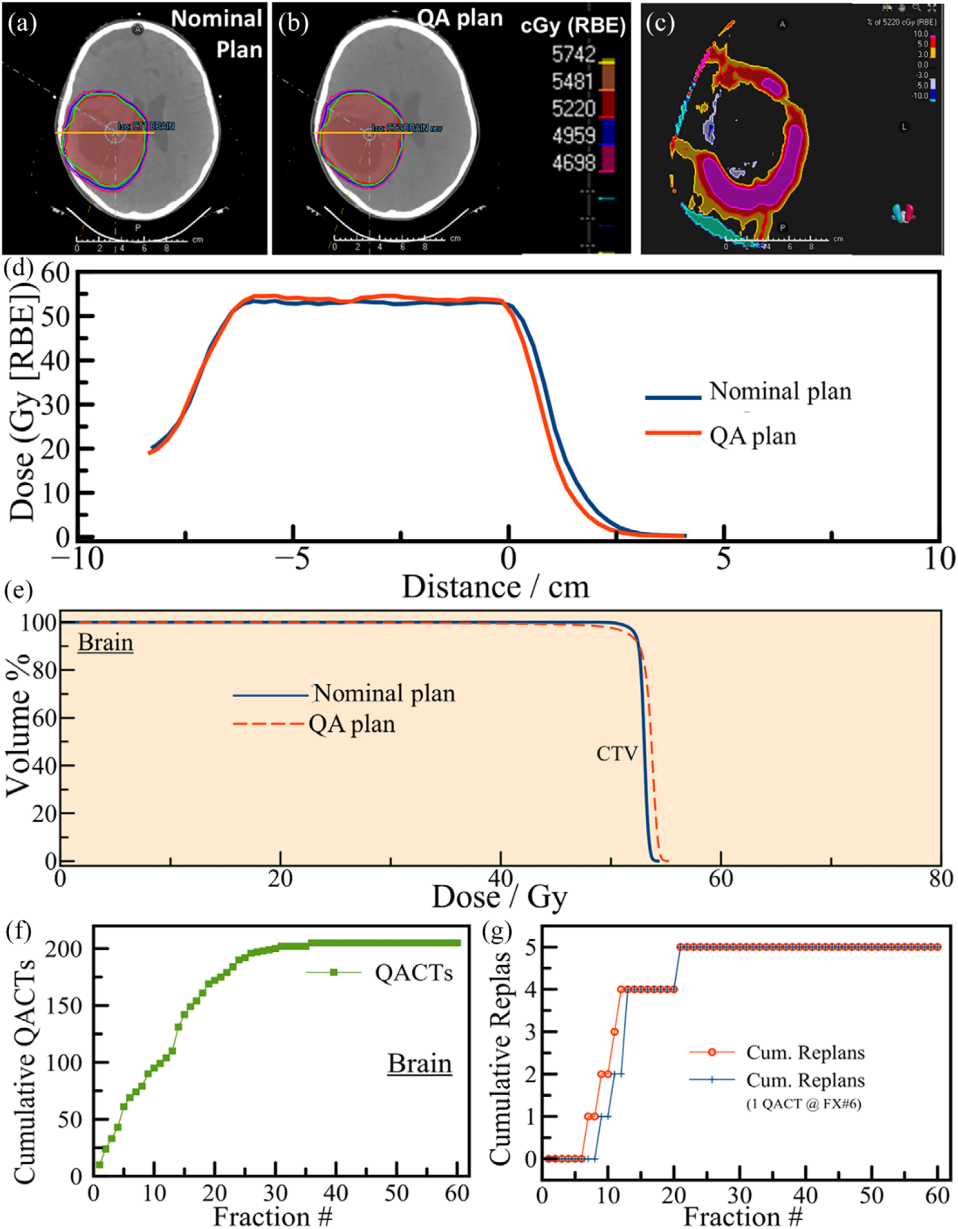
(a) A representative axial slice of the treatment plan of a 7‐year‐old female patient treated with ependymoma of the right temporal‐parietal lobe with a dose of 5220 cGy in 29 fractions. (b) Plan on a QACT at fraction two. (c) Comparison of dose difference between the nominal and QA plans. Due to increased target volume, increased maximum dose, and less target coverage, the patient was adaptively re‐planned. (d) Comparison of dose distribution between the nominal plan and the QA plan along the line drawn in (a) and (b). (e) Dose‐volume histogram (DVH) comparison between the CTVs for the nominal plan and the QA plan. (f) Cumulative QACTs plotted against the treatment fraction number for 205 QACTs of 82 brain cases. (g) Red circles represent the cumulative number of actual replans with fraction numbers, while the blue + symbols represent the cumulative replans based on a proposed method of conducting only one QACT per patient at fraction number 6.

During the study period, 82 patients undergoing treatment for brain cancer were evaluated, with a total of 205 QACT scans conducted, averaging approximately 2.5 scans per patient. A minority, four patients (4.8%), required replanning based on QACT findings, with one patient necessitating a second replan. Consequently, five of the QACT scans (2.4%) directly contributed to revising treatment plans. Out of these, three plans were due to anatomical changes, one with tumor shrinking, and one due to cyst enlargement between simulation and treatment. None of them had concurrent chemotherapy and no prior irradiation. Figure 1a depicts a representative axial slice of a 7‐year‐old female patient treated with ependymoma of the right temporal‐parietal lobe with a dose (5220 cGy/29 FX). At fraction two, a cyst enlargement was detected on QACT between simulation and treatment, resulting in the dose discrepancy as depicted in Figure 1b. The dose difference between the nominal plan (Figure 1a) and the QACT plan (Figure 1b) is depicted in a representative axial slice in Figure 1c. Furthermore, dose differences across a line (yellow, Figure 1a, b) from left to right drawn passing through the tumor isocenter are depicted in Figure 1d and the DVH differences for the CTV are depicted in Figure 1e. Due to this increase in the Dmax (from 104% to 106%), and change in target volume, it was decided to adaptively replan on the QACT. Figure 1f illustrates the cumulative number of QACTs performed across all brain tumor patients as a function of treatment fraction. At each fraction, the plotted value represents the total number of QACTs administered at that specific fraction number across the entire patient cohort. The endpoint of the curve reflects the overall QACT count during the study period, thereby visualizing the temporal distribution of QACT utilization. In contrast, Figure 1g presents the cumulative incidence of adaptive replanning events by treatment fraction. Orange circles denote the actual timing of replans observed during treatment, while blue strikes indicate the hypothetical timing of replans that would have occurred under the proposed QACT scheduling guidelines.

It is evident from Figure 1f that 50% of the QACTs were scanned during the first 2 weeks of treatment. The red circle in Figure 1g represents the revision plan pattern of the 4 patients, with fraction numbers. All first revision plans were delivered within fractions 7 and 12 of the full course of treatment. Furthermore, one patient undergoing two revisions had their initial replan at fraction seventh, followed by a subsequent revision at fraction 21. The interval between conducting QACT scans and the implementation of revision plans ranged from 3 to 7 business days. Given that replanning was necessitated early in the treatment course, it is proposed that a single QACT scan at the start of the second week (by fraction 6) would be optimal. This approach, illustrated by the blue + symbol in Figure 1g, hypothesizes the timing of revision plans if this new QACT scheduling guideline were followed, suggesting potential delays of 1–2 days for some replans. Here, the new timings for the revision plans are the sum of days it had taken to deliver the initial plan and fraction 6 (day of QACT). Also, for those undergoing replanning based on the initial QACT conducted at fraction 6, conducting another QACT at the onset of the fourth week seems beneficial. Implementing this refined scheduling could potentially reduce the annual QACT count by approximately 119 (58% reduction) scans for brain cancer patients at our institution, maintaining treatment quality and accuracy.

### 3.2 Head and neck cancer treatment

**FIGURE 2:**
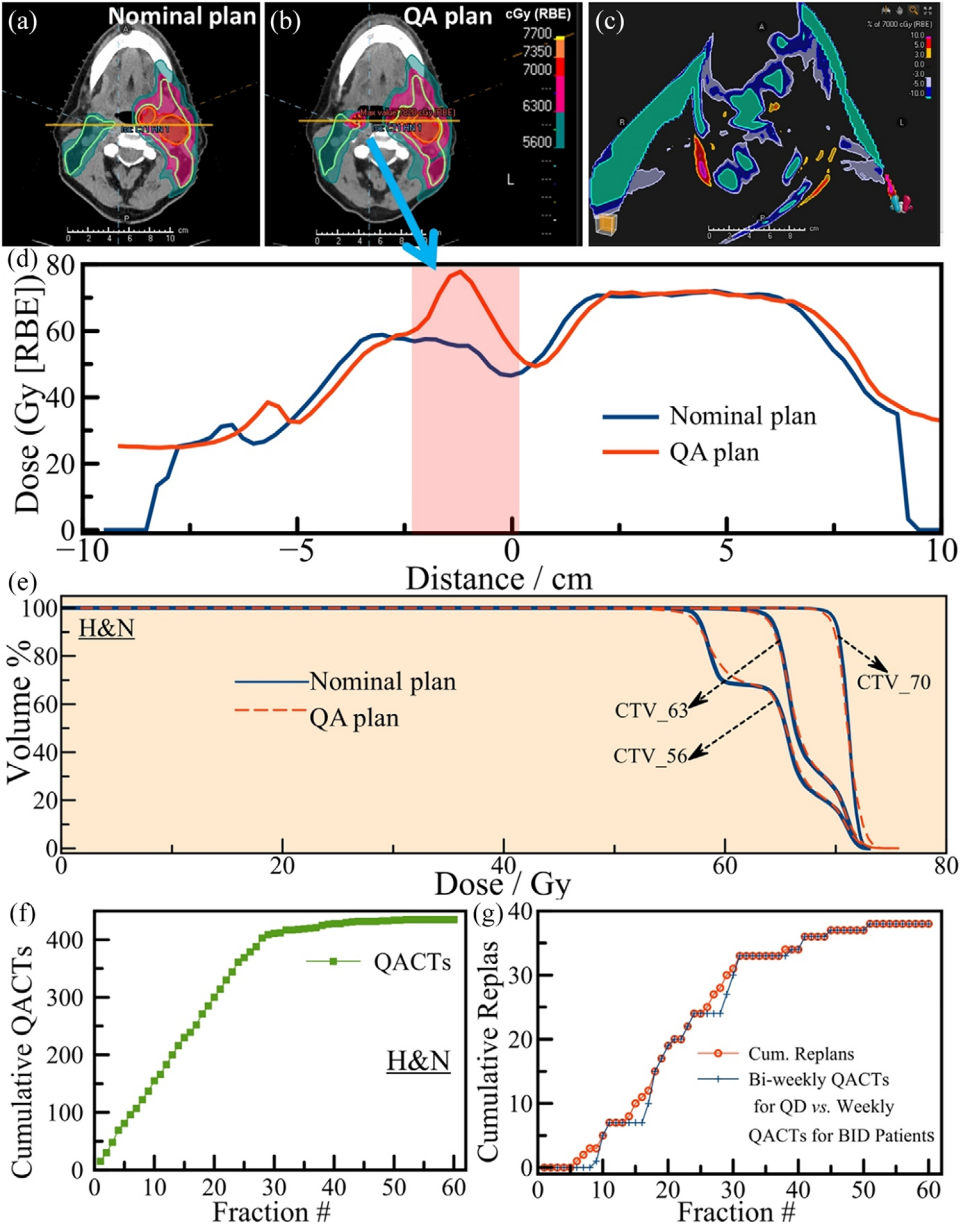
(a) A representative axial slice of the treatment plan of a 65‐year‐old male with a malignant neoplasm of tonsil with a dose of 7000 cGy in 35 fractions. (b) Plan on a QACT at fraction nine. (c) Comparison of dose difference between the nominal and QA plans due to anatomical changes. (d) Comparison of dose distribution between the nominal plan and the QA plan along the line drawn in (a) and (b). (e) DVH comparison between the CTVs (5600, 6300, and 7000 cGy) for the nominal plan and the QA plan. (f) Cumulative QACTs plotted against the treatment fraction number for HN targets. (g) The red circle represents the cumulative number of actual replans with fraction numbers, while the blue + symbols represent replans with only the new QACT pattern, fractions 1 (1–3), 11, 21, 31, 41, etc.

In this study, 97 patients with HN cancers were treated, undergoing a total of 437 QACT scans, averaging about 4.5 scans per patient. The cohort was divided into two treatment schedules: 76 patients received one treatment fraction per day (QD), while 21 patients underwent two fractions per day (BID). A notable portion, 33 patients (34%), required treatment replanning based on QACT outcomes, with five patients undergoing this process twice. Consequently, 38 revision plans were developed from QACTs, accounting for an adaptive replanning rate of 8.7%. Among, 38 revision plans, 14 (36.8%) replans were due to weight loss, 12 (31.6%) due to soft tissue changes, seven (18.4%) of them are due to tumor shrinking, and five (13.1%) due to changes in air cavities near the CTVs. These anatomical changes not only compromised target coverage but also led to increased doses to adjacent OARs, the emergence of new hotspots in critical structures, and a significant rise in the overall maximum dose within the treatment plan. Such dosimetric degradations underscore the importance of incorporating OAR evaluation alongside target coverage in guiding adaptive replanning decisions. Furthermore, among the 33 patients replanned, 25 (75.7%) had concurrent chemotherapy, and eight of the 25 had prior irradiation to the same site. Figure 2a depicts the axial slice of the treatment plan of a 65‐year‐old male with a malignant neoplasm of tonsil (HN patient). Dose to high‐, intermediate‐, and low‐risk targets were prescribed to doses of 7000, 6300, and 5600 cGy in 35 fractions, respectively in a SIB approach, with concurrent chemotherapy. The QACT at the ninth fraction showed the anatomical changes as depicted in Figure 2b, thus triggered the need for adaptive replanning. The dose difference between the nominal plan (Figure 2a) and the QACT plan (Figure 2b) is depicted in a representative axial slice in Figure 2c. Furthermore, dose differences across a line (yellow, Figure 2a, b) from left to right drawn passing through the CTV70 region is depicted in Figure 2d and the corresponding DVH differences for the CTV56, CTV63, and CTV70 are depicted in Figure 2e. Furthermore, Dmax increased beyond 110% and CTVs lost coverages as depicted in Figure 2e, f depicts the distribution of cumulative QACTs across treatment fractions for the HN patient group. As detailed in Figure 2g, revision plans, marked by red circles, began from the sixth fraction onwards, with the interval between QACT and plan revision averaging one week. Within the replanned cohort, 25 patients followed a QD schedule, with three individuals requiring replanning twice. Additionally, eight patients adhered to a BID schedule, and two of the patients underwent replanning twice.

**FIGURE 3:**
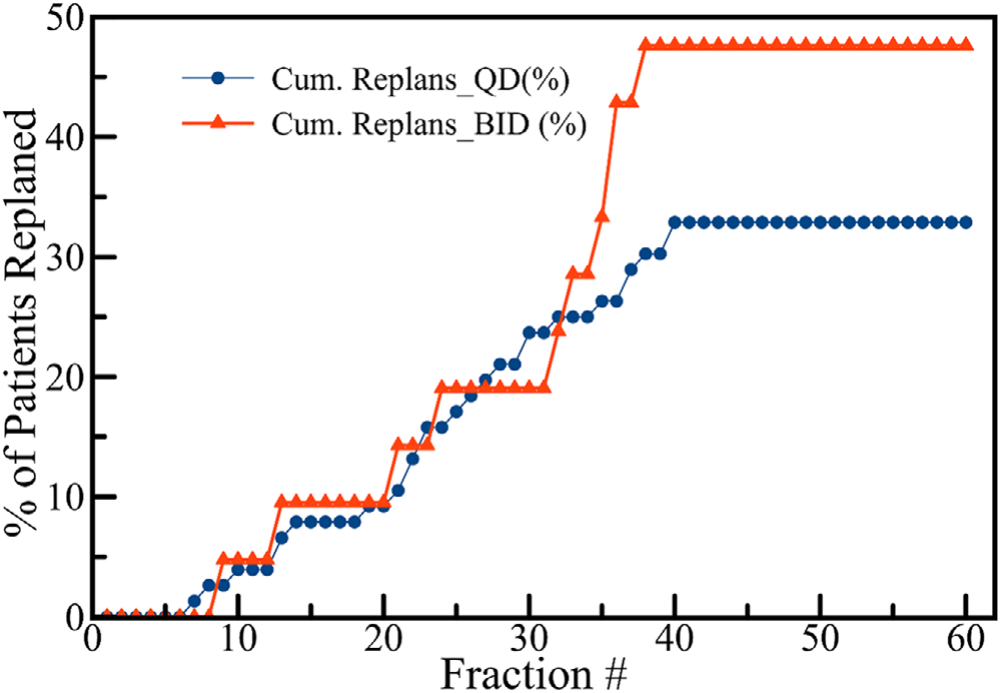
Percentages of patients 76 QD and 21 BID HN patients replanned at the corresponding days after the start of initial treatment.

A revised QACT scheduling strategy was proposed based on the observed replanning patterns, suggesting an initial QACT within the first three fractions, followed by weekly scans for BID patients and bi‐weekly scans for QD patients. This proposed schedule is visualized in Figure 2g with the + symbol, indicating the timing for new QACT scans according to the revised protocol. This adjustment implies that with the new QACT scheduling, all necessary replans could potentially occur within 0–3 days from their originally scheduled dates. Adopting these updated guidelines could lead to a reduction of approximately 127 QACTs (29%) annually for HN patients at our facility, thereby enhancing treatment efficiency without compromising on quality or accuracy. Separate analyses of the replan rates for QD and BID treatment schedules showed that replans for QD patients were initiated between 7 and 40 fractions from the start of treatment. For BID patients, replans were implemented within 9–38 fractions. As depicted in Figure 3, the replan frequencies, represented as percentages, indicate comparable replanning rates between QD and BID schedules.

### 3.3 Prostate cancer treatment

**FIGURE 4:**
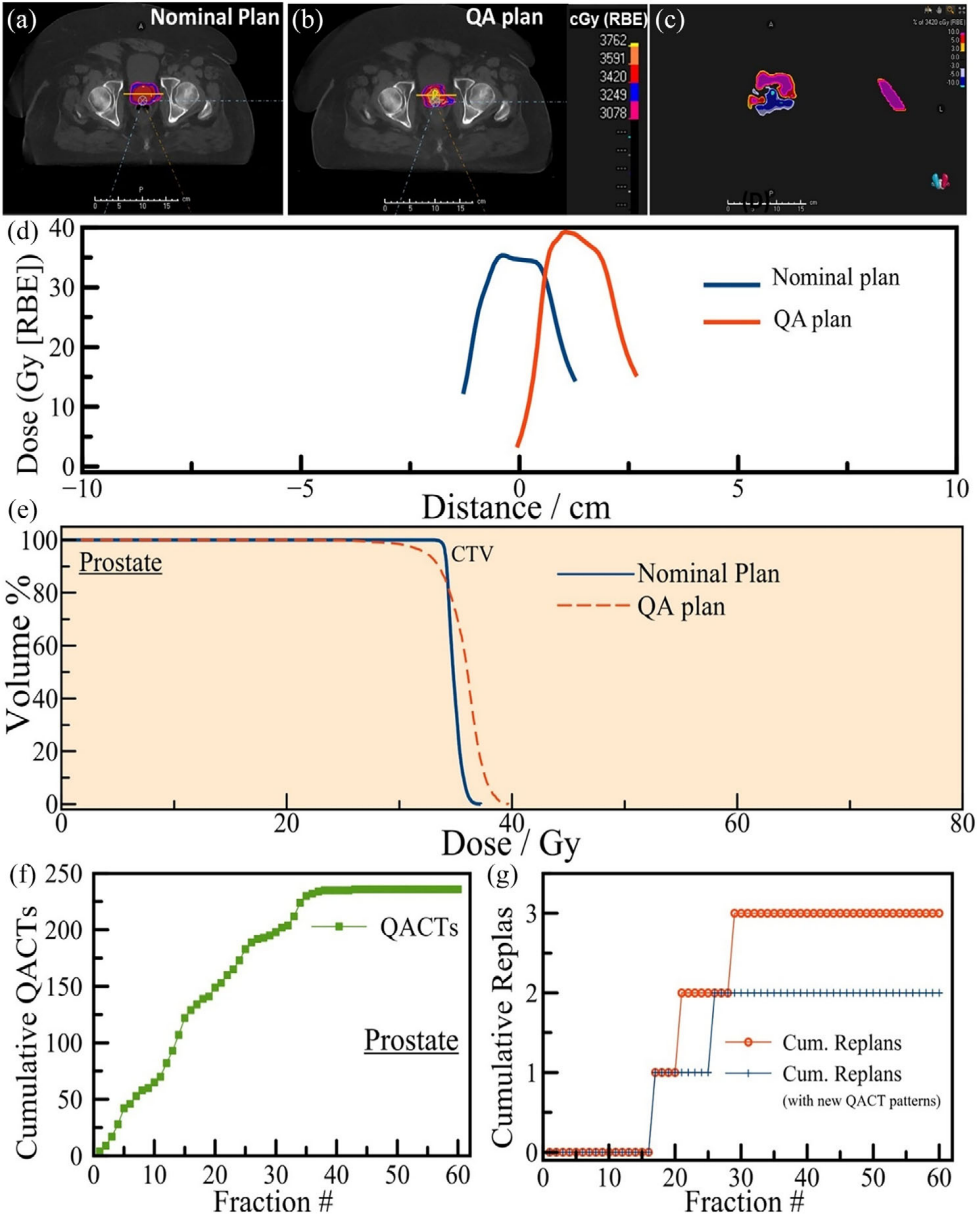
(a) A representative axial slice of the boost treatment plan of a 72‐year‐old patient treated to malignant neoplasm of the prostate with a dose of 4500 cGy in 25 fractions to the whole pelvis followed by a boost of 3420 cGy in 19 fractions to prostate and seminal vesicles. (b) Plan on a QACT at fraction 24. (c) Comparison of dose difference between the two boost plans (a, b) due to anatomical changes. (d) Comparison of dose distribution between the nominal plan and the QA plan along the line drawn in (a) and (b). (e) DVH comparison between the CTVs among the nominal plan and the QA plan. (f) Cumulative QACTs plotted against the treatment fraction number for prostate targets. (g) The Red circle represents the cumulative number of actual replans with fraction numbers, while the blue + symbols represent replans with only new QACT pattern, one QACT/patient for simultaneous integrated boost or treating prostate only, and two QACTs/patient for sequentially planned patients (with prostate, seminal vesicles, and pelvic lymph nodes), as proposed here.

During the specified study period, 84 patients receiving treatment for prostate cancer were assessed. Among these, three patients (3.6%) required treatment replanning based on QACT findings, with a total of 236 QACTs performed, averaging 2.8 scans per patient. Replanning was necessitated in only 1.3% of these QACT scans. A representative axial slice of the boost treatment plan of a 72‐year‐old patient treated to malignant neoplasm of the prostate with a dose of 4500 cGy in 25 fractions to the whole pelvis followed by a boost of 3420 cGy in 19 fractions to prostate and SVs is depicted in Figure 4a. A QACT at fraction 24 did not cover the target because of the anatomical changes as depicted in Figure 4b, hence the plan was adaptively revised on the QACT. The dose difference between the nominal plan (Figure 4a) and the QACT plan (Figure 4b) is depicted in a representative axial slice in Figure 4c. Furthermore, dose differences across a line (yellow, Figure 4a, b) from left to right drawn passing through the CTV region are depicted in Figure 4d and the corresponding DVH differences for the CTV are depicted in Figure 4e. As depicted in Figure 4e, CTV DVH shows a significant loss of coverage and elevated maximum dose, hence triggered for adaptive replan. Also, the distribution of cumulative QACTs among prostate cancer patients is depicted in Figure 4f. Specifically, the three revision plans, indicated by red circles in Figure 4g, were implemented between the 17th and 29th fractions of their treatment schedules. Regarding treatment strategies, the first and third patients underwent sequential planning, while the second patient received a SIB planning approach. An in‐depth review of these three cases revealed significant soft tissue changes along the beam path in the SIB‐planned patient, prompting a revision plan at the 17th fraction as observed on CBCT. The adjustments in the other two patients’ treatment plans were attributed to changes in target coverage and plan robustness, as detected through QACT.

The interval from conducting a QACT to executing a revision plan averaged five business days. Based on an analysis of the QACT and replan patterns, coupled with physician assessments of the overall health status of prostate patients, revised QACT scheduling recommendations were formulated as follows:

1. Prostate‐only patients: Initiate one QACT within the first three fractions of treatment.
2. SIB‐planned patients: Begin with one QACT within the first three fractions, with the possibility of additional scans at the physician's discretion.
3. Sequential planning (initial plan followed by small‐field boost):
a. For patients treated for the prostate, SVs, and pelvic lymph nodes: Schedule one QACT early in the treatment phase and another approximately 1 week before initiating the small‐field boost.
b. For prostate/SV treatment: Recommend one QACT within the first three fractions.

The proposed adjustments are visualized in Figure 4g with blue stars, indicating how the revision plans would align under the new QACT guidelines. Implementing these guidelines could potentially reduce the number of QACTs by approximately 116 scans annually (a 49.1% decrease) for prostate cancer patients at our institution, without compromising treatment quality or accuracy. According to the new QACT scheduling, one patient's replan, necessitated by changes in the robustness of the plan as detected on QACT, might be missed. This case was triggered by CBCT due to significant weight gain, and it would have been caught by the therapists during patient setup.

## 4 DISCUSSION

This study advances the understanding of quality assurance in proton therapy by introducing an evidence‐based, site‐specific framework for optimizing QACT frequency. Utilizing retrospective clinical data, we establish a protocol that strategically balances treatment precision with resource utilization. Given the intrinsic sensitivity of proton therapy to anatomical and range uncertainties, it is inherently less robust than photon‐based modalities. Consequently, even in instances where QACTs do not result in adaptive replanning, they remain indispensable for detecting clinically meaningful dose distribution changes, details that are often undetectable on CBCT due to current limitations in performing accurate dose recalculations on those images. Our analysis reveals a nuanced landscape of adaptive replanning across three critical anatomical sites treated with proton therapy: brain, prostate, and HN cancers. By applying the new QACT program, the brain cases shown in Figure 1 may be determined for replan within 1–3 days later than that would have happened with the original QACTs. For the HN cases shown in Figure 2, with this new QACT pattern, eight replans would have been delayed by 2 days and two replans delayed by 1 day than the original pattern. For the prostate cases, Figure 4, one replan would have been delayed by 3 days and one boost plan would have been missed. The comparative scarcity of morphological and density changes in the vicinity of the brain and prostate lesions accounts for the notably lower replanning rates (1%–2.4%) in contrast to the more dynamic HN region, which exhibits a higher replanning necessity at 8.7%. This variation underscores the importance of tailoring QACT schedules to the specific challenges and stability of each treatment site.

The overarching analysis of our QACT process efficiency indicates that approximately 5% of all QACTs precipitate an adaptive plan, highlighting the precision yet flexibility of our approach. Notably, the absence of replanning needs in the concluding weeks of treatment across all sites suggests an opportunity to streamline the QACT process further by eliminating scans during the last two weeks of therapy. This observation aligns with our strategic adjustment to QACT schedules, aiming to minimize patient exposure to unnecessary imaging and optimize both patient and institutional resources.

To address the potential for variability in anatomical and treatment response, we advocate for an individualized approach to QACT scheduling. Specifically, for patients undergoing significant changes or revisions based on initial QACTs, a more frequent scanning protocol may be warranted, as determined by the treating physician's clinical judgment. Moreover, to enhance our adaptive planning strategy's responsiveness, we recommend an increased frequency of CBCT scans. This adjustment aims to ensure continuous alignment with the highest standards of care, accounting for both anticipated and unforeseen changes during treatment. Concurrent chemotherapy plays an important role in anatomical and tumor changes. Among HN patients, only 42 % (27 of 64) of the non‐replanned group received concurrent chemotherapy, while 75% (25 of 33) of the replanned group received concurrent chemotherapy. Of note, a new planning strategy for bilateral HN cases has been reported, where the plans have been changed from mixed coplanar and noncoplanar beams to coplanar beams only.^23^ In future studies, the impacts of non‐coplanar versus coplanar beams on the rate of replanning need to be investigated.

Following the implementation of revised QACT guidelines in June 2021, our preliminary review over one year demonstrates a promising shift towards efficiency. Notably, the average frequency of QACTs per patient has decreased, leading to more judicious use of imaging resources. The number of QACTs per patient reduced from 2.5 to 1.5 for the brain, 4.5 to 3.2 for HN, and 2.9 to 1.9 for prostate patients. The proportion of QACTs contributing to adaptive replanning has notably increased across all three sites, from 5.2% to 11.3%, indicating a more targeted and potentially impactful utilization of QACTs. For the brain, the percentage of QACT used for adaptive replanning changed from 2.9 % to 10%, for the prostate it changed from 1.3 % to 5.2 %, and for HN patients, it changed from 8.5% to 16.8%. This shift not only exemplifies a more optimal employment of QACTs but also reflects our commitment to enhancing patient experience and resource allocation.

**FIGURE 5:**
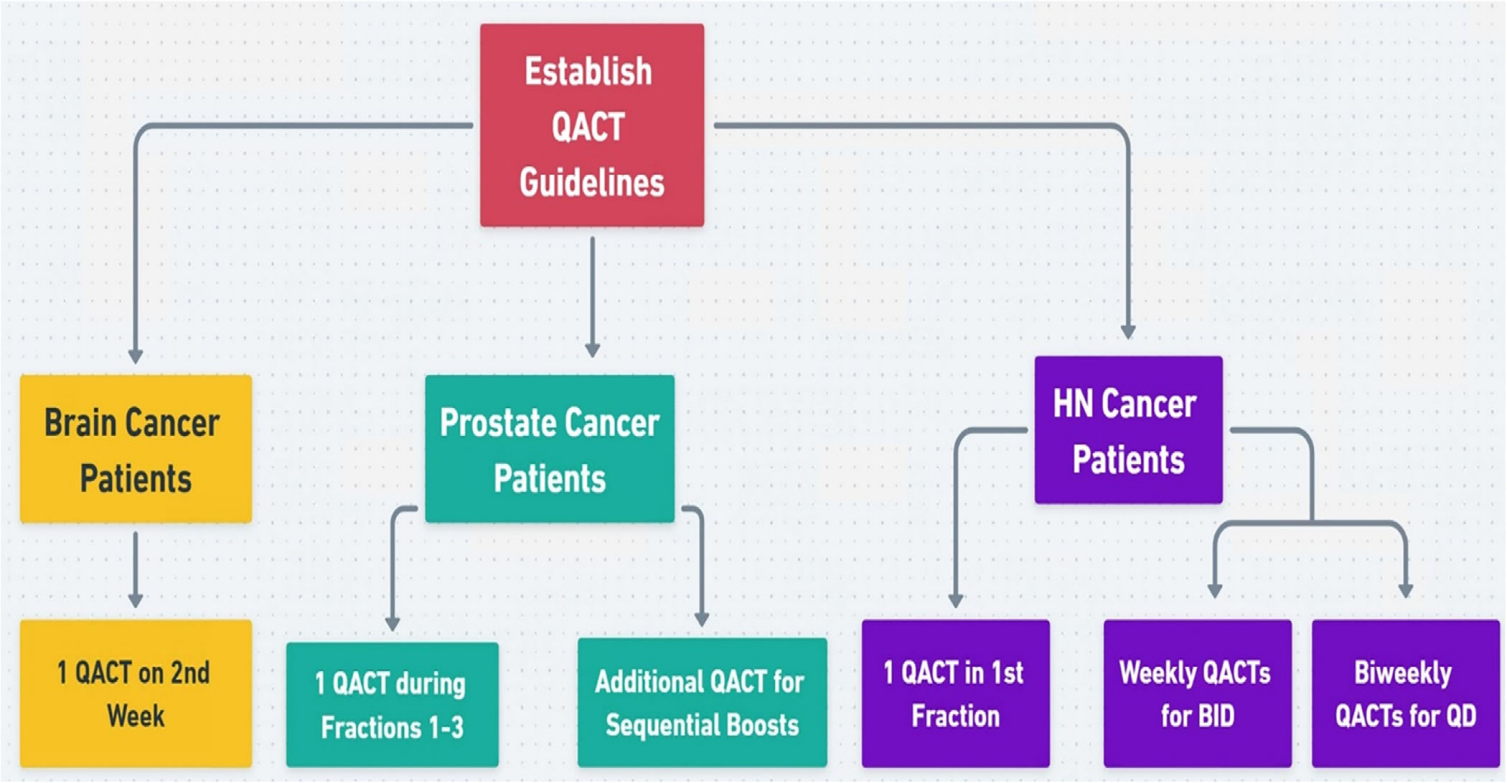
Schematic overview of QACT guidelines for various cancer patient categories, including brain cancer, prostate cancer, and head and neck (HN) cancer patients. The flowchart outlines the established QACT guidelines for each patient group, detailing the recommended timing and frequency of QACTs.

Given the impracticality of daily QACTs in high‐throughput clinics, CBCT imaging serves as an efficient alternative for monitoring bulk anatomical changes and guiding the selective use of QACTs. This strategic integration of imaging modalities ensures adaptive planning remains both clinically effective and resource‐conscious. The implementation of QACT guidelines for various cancer patient categories was systematically outlined in Figure 5. The schematic overview provides a detailed flowchart of the established QACT guidelines, specifying the recommended timing and frequency for brain cancer, prostate cancer, and HN cancer patients at different stages, highlighting the specific considerations and adjustments made for each patient group. Furthermore, these modifications in the QACT strategy underscore a broader commitment to adaptive and patient‐centered care. By aligning QACT frequency with the dynamic nature of tumor and tissue responses, we aim to balance the imperative of meticulous quality assurance with the practicalities of clinical efficiency and patient well‐being. This delicate balance between rigor and flexibility in proton therapy underscores our ongoing quest to refine treatment protocols in pursuit of optimal outcomes. Ultimately, periodic monitoring using QACTs needs to be replaced with that using CBCTs, as we are practicing for photon treatments. In a recent study, Yao et al. demonstrated that a routine scheduled QACT program for HN proton treatments may be replaced with QACTs only when needed if daily CBCTs are properly used to determine the water equivalent thickness of the beam tracks.^24^ Biswal et al. also demonstrated that routine QACT can be replaced by monitoring morphologic change with daily CBCTs.^25^ As we analyze the impact of these guideline adjustments, we remain attentive to opportunities for further enhancing the precision and patient‐centric nature of our treatment strategies.

Given the impracticality of daily QACTs in high‐throughput clinics, CBCT imaging serves as an efficient alternative for monitoring bulk anatomical changes and guiding the selective use of QACTs. This strategic integration of imaging modalities ensures adaptive planning remains both clinically effective and resource‐conscious.

Furthermore, Evans et al. suggested weekly verification scans, citing substantial inter‐fraction variability and range uncertainties in HN tumors, which can jeopardize OAR sparing and target coverage. Similarly, Kraan et al. quantified dose degradation due to anatomical, range, and setup errors across 3700 simulations for oropharyngeal cancer patients, reporting that only 69% of plans met the clinical D98% ≥ 95% threshold without adaptation, whereas adaptive planning increased this to 96%, reinforcing the value of periodic QACTs. Our retrospective analysis observed a 37.1% replan rate in HN patients compared to 3.1% and 1.4% in brain and prostate patients, respectively, confirming the greater anatomical variability in the HN region. However, by aligning QACT frequency with empirically derived replan intervals, our every‐other‐week protocol maintains 97% replan capture within a 3‐day buffer, demonstrating a more resource‐efficient yet clinically effective strategy than weekly scans. While this study provides general guidelines for patients across the three anatomical sites (brain, HN, and prostate), clinicians may still request more frequent QACTs at their discretion for (i) re‐irradiation cases, (ii) patients in whom OAR sparing is prioritized over tumor coverage, and (iii) situations where significant changes in fluid or air cavities are expected. This provides an opportunity to further extend our framework to define QACT frequency guidelines across all major anatomical treatment sites. Concurrently, we are developing standardized, evidence‐based criteria for evaluating QACTs to systematically identify cases that caution adaptive replanning interventions.

## 5 CONCLUSION

Patterns of replanning for three anatomic sites (brain, HN, and prostate) were retrospectively analyzed to establish guidelines for QACT patterns during the course of treatment. For brain patients, one QACT per patient at the beginning of the second week (fraction 6) of treatment was suggested. One QACT at the first fraction (or no later than the third fraction) and then weekly for BID patients and biweekly for QD patients was suggested for HN patients. Similarly, for prostate patients, one QACT was recommended during fractions 1–3, and one more QACT was recommended for those patients whose treatment involves the prostate with SVs and lymph nodes and planned as sequential boosts. For all the sites, a QACT is warranted on the first day of the treatment if the gap between simulation and treatment exceeds more than three weeks. With this new proposed pattern, the number of QACTs could be reduced from 205 to 86 (58% reduction) for the brain, 437 to 310 (29.1% reduction) for HN, and 236 to 120 (49.1% reduction) for prostate patients. A total of 878 QACTs could be reduced to 517, a 41% reduction for these three sites together. Post‐implementation of these new guidelines, it was found that the percentage of QACTs used for adaptive replanning changed from 5.2% to 11.3% for the three sites together.

## AUTHOR CONTRIBUTIONS

**Nrusingh C. Biswal**: Conceptualization; data collection and Curation; formal analysis; investigation; methodology; project administration; resources; validation; visualization; writing—original draft; review; and editing. **Mark J. Zakhary**: Formal analysis; review; and editing. **Abdul K. Parchur**: Formal analysis; review; and editing. **Ruslan Mogilnay**: Data Curation; scripting. **Matthew J. Ferris**: Resources; supervision; review; and editing. **Elizabeth M. Nichols**: Project administration; resources; supervision; visualization. **Matthew E. Witek**: Project administration; resources; supervision; review; and editing. **ByongYong Yi**: Idea Development; formal analysis; project administration; investigation; methodology; resources; supervision; review; and editing.

## CONFLICT OF INTEREST STATEMENT

The authors declare no conflicts of interest.

## Data Availability

The data that support the findings of this study are available from the corresponding author upon reasonable request.

## References

[1] Zhang X , Dong L , Lee AK , et al. Effect of anatomic motion on proton therapy dose distributions in prostate cancer treatment. Int J Radiat Oncol Biol Phys. 2007;67:620‐629. 17236979 10.1016/j.ijrobp.2006.10.008 PMC1945214

[2] Hui Z , Zhang X , Starkschall G , et al. Effects of interfractional motion and anatomic changes on proton therapy dose distribution in lung cancer. Int J Radiat Oncol Biol Phys. 2008;72:1385‐1395. 18486357 10.1016/j.ijrobp.2008.03.007 PMC3401022

[3] Kraan AC , van de Water S , Teguh DN , et al. Dose uncertainties in IMPT for oropharyngeal cancer in the presence of anatomical, range, and setup errors. Int J Radiat Oncol Biol Phys. 2013;87:888‐896. 24351409 10.1016/j.ijrobp.2013.09.014

[4] Zhang Y , Alshaikhi J , Amos RA , et al. Improving workflow for adaptive proton therapy with predictive anatomical modelling: a proof of concept. Radiother Oncol. 2022;173:93‐101. 35667573 10.1016/j.radonc.2022.05.036

[5] Schaly B , Kempe J , Venkatesan V , Mitchell S , Chen J . Alert system for monitoring changes in patient anatomy during radiation therapy of head and neck cancer. J Appl Clin Med Phys. 2021;22:168‐174. 10.1002/acm2.13342 PMC8364268 34302421

[6] Jagt TZ , Breedveld S , van Haveren R , Heijmen BJM , Hoogeman MS . Online‐adaptive versus robust IMPT for prostate cancer: how much can we gain? Radiother Oncol. 2020;151:228‐233. 32777242 10.1016/j.radonc.2020.07.054

[7] Wu RY , Liu AY , Sio TT , et al. Intensity‐modulated proton therapy adaptive planning for patients with oropharyngeal cancer. Int J Part Ther. 2017;4:26‐34. 31773006 10.14338/IJPT-17-00010.1 PMC6871549

[8] Schaly B , Kempe J , Venkatesan V , Mitchell S , Battista JJ . Using gamma index to flag changes in anatomy during image‐guided radiation therapy of head and neck cancer. J Appl Clin Med Phys. 2017;18:79‐87. 28901659 10.1002/acm2.12180 PMC5689936

[9] Yao W , Krasin MJ , Farr JB , Merchant TE . Feasibility study of range‐based registration using daily cone beam CT for intensity‐modulated proton therapy. Med Phys. 2018;45:1191‐1203. 29360157 10.1002/mp.12760

[10] Bohannon D , Janopaul‐Naylor J , Rudra S , et al. Prediction of plan adaptation in head and neck cancer proton therapy using clinical, radiographic, and dosimetric features. Acta Oncol. 2023;62:627‐634. 37335043 10.1080/0284186X.2023.2224050

[11] Yao W , Schweitzer N , Biswal N , Polf J , Farr J , Vujaskovic Z . Impact of bowel and rectum air on target dose with robustly optimized intensity‐modulated proton therapy plans. Acta Oncol. 2020;59:1186‐1192. 32500780 10.1080/0284186X.2020.1769859

[12] Hedrick SG , Fagundes M , Case S , et al. Validation of rectal sparing throughout the course of proton therapy treatment in prostate cancer patients treated with SpaceOAR((R)). J Appl Clin Med Phys. 2017;18:82‐89. 28291933 10.1002/acm2.12010 PMC5689883

[13] Yao W , Zhang B , Han D , et al. Use of CBCT plus plan robustness for reducing QACT frequency in intensity‐modulated proton therapy: head‐and‐neck cases. Med Phys. 2022;49:6794‐6801. 35933322 10.1002/mp.15915

[14] Nenoff L , Matter M , Amaya EJ , et al. Dosimetric influence of deformable image registration uncertainties on propagated structures for online daily adaptive proton therapy of lung cancer patients. Radiother Oncol. 2021;159:136‐143. 33771576 10.1016/j.radonc.2021.03.021

[15] Nesteruk KP , Bobic M , Lalonde A , Winey BA , Lomax AJ , Paganetti H . Ct‐on‐rails versus in‐room cbct for online daily adaptive proton therapy of head‐and‐neck cancers. Cancers (Basel). 2021;1‐13. 34885100 10.3390/cancers13235991 PMC8656713

[16] Veiga C , Janssens G , Teng CL , et al. First clinical investigation of cone beam computed tomography and deformable registration for adaptive proton therapy for lung cancer. Int J Radiat Oncol Biol Phys. 2016;95:549‐559. 27084664 10.1016/j.ijrobp.2016.01.055

[17] Kurz C , Nijhuis R , Reiner M , et al. Feasibility of automated proton therapy plan adaptation for head and neck tumors using cone beam CT images. Radiat Oncol. 2016;11:64. 27129305 10.1186/s13014-016-0641-7 PMC4851791

[18] Ger RB , Sheikh K , Gogineni E , et al. Planning and treatment recommendations for breast proton therapy from a single center's experience. Adv Radiat Oncol. 2023;8:101069. 36213549 10.1016/j.adro.2022.101069 PMC9535282

[19] Ding X , Younkin JE , Shen J , Bues M , Liu W . A critical review of the practices of proton daily quality assurance programs. Ther Radiol Oncol. 2021;5:1‐17.

[20] Evans JD , Harper RH , Petersen M , et al. The importance of verification ct‐qa scans in patients treated with impt for head and neck cancers. Int J Part Ther. 2020;7:41‐53. 33094135 10.14338/IJPT-20-00006.1 PMC7574830

[21] Nichols EM , Regine WF , Simone CB , Langen KM . Single‐institutional experience assessing the role of quality assurance (qa) ct scans and their impact on adaptive planning with pencil‐beam scanning proton therapy in the setting of cone beam ct (cbct). Int J Radiat Oncol *Biol Phys*. 2018;102:e539.

[22] Biswal NC , Rodrigues DB , Yao W , Molitoris JK , Witek ME , Chen S . Evaluation of intrafraction couch shifts for proton treatment delivery in head‐and‐neck cancer patients: toward optimal imaging frequency. J Appl Clin Med Phys. 2022;23:e13795. 36239306 10.1002/acm2.13795 PMC9797163

[23] Yi B , Zatczak J , Deng W , et al. Is noncoplanar plan more robust to inter‐fractional variations than coplanar plan in treating bilateral HN tumors with pencil‐beam scanning proton beams?. J Appl Clin Med Phys. 2024;25:e14186(1‐9). 37974385 10.1002/acm2.14186 PMC10860533

[24] Yao W , Zhang B , Han D , et al. Use of CBCT plus plan robustness for reducing QACT frequency in intensity‐modulated proton therapy: head‐and‐neck cases. Med Phys. 2022;49(11):6794‐801. 35933322 10.1002/mp.15915

[25] Biswal NC , Zhang B , Nichols E , Witek ME , Regine WF , YI B . Cone‐beam ct images as an indicator of qact during adaptive proton therapy of extremity sarcomas. Int J Part Ther. 2024;12:100017 (1‐7). 39022119 10.1016/j.ijpt.2024.100017 PMC11252065

